# A simple risk-based strategy for hepatitis C virus screening among incarcerated people in a low- to middle-income setting

**DOI:** 10.1186/s12954-020-00400-4

**Published:** 2020-08-14

**Authors:** Sanam Hariri, Maryam Sharafkhah, Maryam Alavi, Gholamreza Roshandel, Abdolreza Fazel, Taghi Amiriani, Nazgol Motamed-Gorji, Abolfazl Bazazan, Shahin Merat, Hossein Poustchi, Reza Malekzadeh

**Affiliations:** 1 Liver and Pancreatobiliary Diseases Research Center, Digestive Diseases Research Institute, Shariati Hospital, Tehran University of Medical Sciences, Tehran, Iran; 2 Digestive Disease Research Center, Digestive Disease Research Institute, Shariati Hospital, Tehran University of Medical Sciences, Tehran, Iran; 3 The Kirby Institute, UNSW Sydney, Sydney, NSW Australia; 4 Golestan Research Center of Gastroenterology and Hepatology, Golestan University of Medical Sciences, Gorgan, Iran; 5 Cancer Research Center, Golestan University of Medical Sciences, Gorgan, Iran; 6 Department of Health, Golestan State Prisons and Security and Corrective Measures Organization, Gorgan, Iran; 7 Digestive Oncology Research Center, Digestive Diseases Research Institute, Shariati Hospital, Tehran University of Medical Sciences, Tehran, Iran

**Keywords:** Hepatitis C virus (HCV), Screening, Micro-elimination, Prison healthcare, Harm reduction, Drug use, Opioid agonist therapy, Low- and middle-income countries

## Abstract

**Background:**

Hepatitis C virus (HCV) is among the highest priority diseases in custodial settings; however, the diagnosis remains suboptimal among people in custody. This study aimed to validate a short survey for identifying people with HCV infection in a provincial prison in Iran.

**Methods:**

Between July and December 2018, residents and newly admitted inmates of Gorgan central prison completed a questionnaire, including data on the history of HCV testing, drug use, injecting drug use, sharing injecting equipment, and imprisonment. Participants received rapid HCV antibody testing, followed by venipuncture for RNA testing (antibody-positive only). Each enrollment question (yes/no) was compared with the testing results (positive/negative).

**Results:**

Overall, 1892 people completed the questionnaire, including 621 (34%) who were currently on opioid agonist therapy (OAT); 30% of participants had been tested for HCV previously. About 71% had a history of drug use, of whom 13% had ever injected drugs; 52% had ever shared injecting equipment. The prevalence of HCV antibody and RNA was 6.9% (*n* = 130) and 4.8% (*n* = 90), respectively. The antibody prevalence was higher among people on OAT compared to those with no history of OAT (11.4% vs. 4.0%). History of drug use was the most accurate predictor of having a positive HCV antibody (sensitivity: 95.2%, negative predictive value: 98.9%) and RNA testing (sensitivity: 96.7%, negative predictive value: 99.5%). The sensitivity of the drug use question was lowest among people with no OAT history and new inmates (87% and 89%, respectively). Among all participants, sensitivity and negative predictive value of the other questions were low and ranged from 34 to 54% and 94 to 97%, respectively.

**Conclusions:**

In resource-limited settings, HCV screening based on having a history of drug use could replace universal screening in prisons to reduce costs. Developing tailored screening strategies together with further cost studies are crucial to address the current HCV epidemic in low- to middle-income countries.

## Background

Hepatitis C virus (HCV) infection is a global health concern with a rising disease burden [1]. The introduction of highly effective therapies has revolutionized the treatment landscape, particularly in low- and middle-income countries (LMIC) [2]. However, HCV diagnosis remains suboptimal in many regions, including Iran [3]. To achieve the World Health Organization’s (WHO) target of HCV elimination as a public health issue by 2030, we need effective screening and diagnosis strategies among high-risk groups, including people in custody [1, 4].

In Iran, the prevalence of HCV infection among the general population is estimated at 0.6% [5]. However, prevalence is approximately 45% among people who inject drugs (PWID), 28% among people in prison, and 53% among prisoners with a history of drug injection [6–9]. Generic direct-acting antivirals (DAA) with more than 95% cure rates in 12 weeks of therapy have been formulated in Iran and are available at low prices [10–12]. Despite the potential for treatment with short-duration regimens, HCV diagnosis and treatment uptake have been low among people in custody [4]. In the last decade, in Iranian prisons, harm reduction initiatives, including opioid agonist therapy (OAT), have been widely implemented [13, 14]. Further, the diagnosis and treatment of infectious diseases, including TB and HIV, have significantly increased [15, 16]. The existing infrastructure of harm reduction and primary health care could be utilized to increase HCV diagnosis and linkage to care as well, given the development of low-cost methods for active case finding [17].

In high-income countries, risk-based surveys have been used to increase HCV diagnosis in custodial settings [18, 19]. However, there is no information on previous risk-based screening for infectious diseases in LMIC. Therefore, the effectiveness of this method for enhancing the HCV diagnosis among marginalized populations is questioned. In a large provincial prison with no HCV programs, we aimed to validate a 1-min survey vs. HCV antibody and RNA testing for the identification of people with infection.

## Methods

### Study population and design

This study was a non-randomized trial, evaluating the reliability of a self-reported questionnaire in predicting HCV antibody and RNA testing results. Enrollment occurred between July and December 2018. The study site was Gorgan central prison, located in Northern Iran. All residents and newly admitted inmates were eligible to participate in the study, given they were at least 18 years old and had provided written consent. Participation was voluntary, and participants could withdraw their consent at any time. The Institutional Review Board of Tehran University of Medical Sciences approved the research protocol.

### Study site

Gorgan central prison is located in Gorgan city, Golestan province, Northern Iran. This prison has an approximate inmate population of 2000 and consists of eight wards: public, remands, serious crimes, female, juvenile, solitary confinement, and two wards for people receiving OAT. The prison had a general practitioner and two nurses providing healthcare services, including harm reduction activities and HIV testing. However, there was no HCV program available in this prison.

### Study procedures

Before enrollment, prison health officials invited all inmates to provide education on HCV disease and its complications, routes of transmission, and safe injection techniques. Further, an illustrated booklet was given to participants, containing useful information about HCV infection and liver health. Residents and newly admitted inmates completed a short questionnaire, including information on the history of HCV testing, drug use, injecting drug use, sharing injecting equipment, and imprisonment (Table 1).

**Table 1:** Screening questionnaire of hepatitis C risk factors in the Gorgan prison study

Question	Answer
1. Have you ever been tested for hepatitis C?	A. Yes, I have been tested. Currently, I do not have hepatitis C. B. Yes, I have been tested. Currently, I have hepatitis C. C. Yes, I have been tested. Currently, I do not know whether I have hepatitis C or not. D. No, I have not been tested. E. I do not know.
2. Do you have a history of drug use, ever?	A. Yes B. No
3. Do you have a history of injection drug use, ever?	A. Yes B. No
4. Do you have a history of sharing injection equipment, ever? (Including needle, syringe, spoon, solvent, filter or water)	A. Yes B. No
5. Do you have a history of prior imprisonment? (Only newly admitted inmates)	A. Yes B. No

All participants received on-site rapid HCV antibody testing, using a blood specimen from a fingerstick; post-test counseling was provided. Among participants with a positive HCV antibody test, the prison nurses were available to collect venipuncture blood samples weekly. These participants were invited to attend the prison clinic once a week to provide a sample for HCV RNA testing and other routine clinical care, including HIV and hepatitis B virus serology, liver function tests, and complete blood count.

Participants with positive HCV RNA received treatment with a locally manufactured combination of 400 mg Sofosbuvir and 60 mg Daclatasvir (Sovodak®). AST to Platelet Ratio Index (APRI) was used for liver disease assessment. The duration of antiviral therapy ranged from 12 to 24 weeks for people without cirrhosis (APRI < 1) and those with cirrhosis (APRI > 1), respectively. We delivered all required education for treatment and monitoring to the prison physician. Patients released during the study were referred to the local health network for follow-up. All study activities were provided to participants free of charge.

### Study outcomes

The primary outcome of this study was to investigate the role of a 1-min questionnaire as a risk-based strategy in HCV screening. The other outcome was to evaluate the prevalence of HCV antibody and HCV RNA among all participants and people with a positive antibody test, respectively.

### Statistical analysis

Data are presented as median (interquartile range (IQR)) or frequency and percentage, as appropriate. Each enrollment questionnaire (yes/no) was compared with the results of the HCV antibody testing (positive/negative) and RNA testing (positive/negative). The RNA testing results were evaluated among participants with available information. The enrollment question about previous HCV testing had multiple answers; for comparison, we combined the first three answers (Table 1). Sensitivity, specificity, positive predictive value (PPV), and negative predictive value (NPV) of each question were estimated.

## Results

### Study population

Overall, 1892 participants were enrolled, including 1482 residents with a median age of 35 years (IQR 29–41) and 410 newly admitted inmates*.* The majority were male (96%), did not have higher education (89%), had a monthly income at minimum wage or below (77%), and 34% were currently receiving OAT services. Residents had lower education and monthly income, compared to newly admitted inmates. Similarly, people who were receiving OAT had lower education and monthly income than those who were not currently on OAT (Tables 2 and 3).

**Table 2:** Frequency of risk behaviors and HCV screening among Gorgan prison residents and new inmates, *n* = 1892

	Residents	New inmates	All
Characteristics, *n* (%)	*n* = 1482	*n* = 410	*n* = 1892
Age, median (IQR)	35 (29–41)	–	35 (29–41)
Sex			
Male	1414 (95.4%)	410 (100%)	1824 (96.4%)
Female	68 (4.6%)	–	68 (3.6%)
Highest educational level			
Did not go to school	143 (9.7%)	25 (6.1%)	168 (8.9%)
Did not finish high school	911 (61.5%)	161 (39.3%)	1072 (56.7%)
Finished high school	268 (18.1%)	172 (42.0%)	440 (23.3%)
Higher education	112 (7.6%)	47 (11.5%)	159 (8.4%)
Missing	48 (3.2%)	5 (1.2%)	53 (2.8%)
Monthly income			
Minimum wage or below	1194 (80.6%)	271 (66.1%)	1465 (77.4%)
Living wage	185 (12.5%)	104 (25.4%)	289 (15.3%)
Above living wage	65 (4.4%)	25 (6.1%)	90 (4.8%)
Missing	38 (2.6%)	10 (2.4%)	48 (2.5%)
Drug use, ever			
No	419 (28.3%)	128 (31.2%)	547 (29.0%)
Yes	1059 (71.5%)	282 (68.8%)	1341 (70.9%)
Injecting drug use, ever			
No	910 (85.9%)	254 (90.1%)	1,164 (86.8%)
Yes	146 (13.8%)	28 (9.9%)	174 (13.0%)
Shared injection equipment, ever			
No	62 (42.5%)	10 (35.7%)	72 (41.4%)
Yes	73 (50.0%)	18 (64.3%)	91 (52.3%)
Missing	11 (7.5%)	0 (0.0%)	11 (6.3%)
Imprisonment, ever			
No	–	128 (31.2%)	128 (31.2%)
Yes	–	282 (68.8%)	282 (68.8%)
HCV screening, ever			
No	874 (59.0%)	342 (83.4%)	1216 (64.3%)
Do not know	77 (5.2%)	36 (8.8%)	113 (6.0%)
Yes, currently have HCV	21 (1.4%)	3 (0.7%)	24 (1.3%)
Yes, currently do not have HCV	191 (12.9%)	14 (3.4%)	205 (10.8%)
Yes, do not know the current status	315 (21.3%)	14 (3.4%)	329 (17.4%)

*IQR* interquartile range

**Table 3:** Frequency of risk behaviors and HCV screening categorized by history of opioid agonist therapy (OAT)

	People on OAT	People with a history of OAT	People with no history of OAT
Characteristics, *n* (%)	*n* = 621	*n* = 241	*n* = 949
Sex			
Male	602 (96.9%)	237 (98.3%)	909 (95.3%)
Female	19 (3.1%)	4 (1.7%)	45 (4.7%)
Highest educational level			
Did not go to school	62 (10.0%)	12 (5.0%)	86 (9.1%)
Did not finish high school	356 (57.3%)	148 (61.4%)	521 (54.9%)
Finished high school	137 (22.1%)	55 (22.8%)	238 (25.1%)
Higher education	47 (7.6%)	18 (7.5%)	82 (8.6%)
Missing	19 (3.1%)	8 (3.3%)	22 (2.3%)
Monthly income			
Minimum wage or below	508 (81.8%)	191 (79.3%)	709 (74.7%)
Living wage	87 (14.0%)	33 (13.7%)	158 (16.7%)
Above living wage	21 (3.4%)	9 (3.7%)	51 (5.4%)
Missing	5 (0.8%)	8 (3.3%)	31 (3.3%)
Drug use, ever			
No	52 (8.4%)	66 (27.4%)	390 (41.1%)
Yes	569 (91.6%)	174 (72.2%)	557 (58.7%)
Injecting drug use, ever			
No	468 (82.3%)	153 (87.9%)	506 (90.8%)
Yes	100 (17.6%)	21 (12.1%)	49 (8.8%)
Shared injection equipment, ever			
No	35 (35.0%)	9 (42.7%)	26 (53.1%)
Yes	57 (57.0%)	11 (52.4%)	21 (42.7%)
Missing	8 (8.0%)	1 (4.8%)	2 (4.1%)
HCV screening, ever			
No	395 (63.6%)	142 (58.9%)	622 (65.5%)
Do not know	24 (3.9%)	16 (6.6%)	71 (7.5%)
Yes, currently have HCV	12 (1.9%)	3 (1.2%)	8 (0.8%)
Yes, currently do not have HCV	54 (8.7%)	34 (14.1%)	107 (11.3%)
Yes, do not know the current status	136 (21.9%)	45 (18.7%)	137 (14.4%)

Among all participants, 71% (*n* = 1341) had a history of drug use, of whom 13% (*n* = 174) had a history of injecting drug use; 52% (*n* = 91) of people with injecting drug use had ever shared injecting equipment. The history of drug use and injecting among residents was slightly higher than new inmates (72% vs. 69%, and 14% vs. 10%). People who were currently receiving OAT had a higher prevalence of drug use, injecting drug use, and sharing injecting equipment, compared to those who were not currently on OAT (92% vs. 62%; 18% vs. 10%, and 57% vs. 48%, respectively) (Table 3).

### History of HCV testing

Overall, 30% (558/1887) of participants had a history of HCV testing, including 36% (527/1478) and 8% (31/409) among residents and newly admitted inmates, respectively. Among people who had a history of HCV testing, only 41% (229/558) were aware of their test results. Having a history of testing was reported in 33% and 28% of participants on OAT and those who were not currently on OAT, respectively (Tables 2 and 3).

### Prevalence of HCV antibody and RNA

HCV antibody was detected in 6.9% (*n* = 130) of all participants, including 7.5% (*n* = 111) of residents and 4.6% (*n* = 19) of newly admitted inmates. Among residents, the prevalence of HCV antibody was highest in OAT wards with 13.2% (80/607), followed by remands 3.5% (8/230) and public 3.5% (11/317). The prevalence of HCV RNA among residents was 5.7% (*n* = 84). Out of 19 newly admitted inmates with a positive antibody in the remand ward, 11 were released before the RNA testing; among those who received venipuncture, the HCV viremic rate was 75% (6 of 8). For participants who were currently on OAT and those who were not receiving OAT, the prevalence of antibody was 11.4% (71/621) and 4.6% (55/1190); HCV RNA was detected in 8.7% (54/619) and 2.9% (34/1182), respectively (Table 4).

**Table 4:** Prevalence of HCV antibody and HCV RNA among Gorgan prison participants

	Total	Residents	New inmates	People on OAT	People with a history of OAT	People with no history of OAT
Prevalence, *n* (%)	*n* = 1892	*n* = 1482	*n* = 410	*n* = 621	*n* = 241	*n* = 949
HCV antibody	130 (6.9%)	111 (7.5%)	19 (4.6%)	71 (11.4%)	17 (7.1%)	38 (4.0%)
HCV RNA	90 (4.8%)	84 (5.7%)	6 (1.5%)	54 (8.7%)	10 (4.2%)	24 (2.5%)

*OAT* opioid agonist therapy

### Concordance of the risk-based questionnaire and antibody testing

The drug use question was the most accurate predictor of having a positive HCV antibody test (sensitivity: 95.4%, negative predictive value: 98.9%), with a higher sensitivity in residents compared to new inmates (96% vs. 89%). The sensitivity of the drug use question among participants who were currently receiving OAT and those with and without a history of OAT were 100%, 94%, and 87%, respectively (Tables 5 and 6).

**Table 5:** Characteristics of the questionnaire for detecting HCV antibody among Gorgan prison residents and new inmates

History of risk behaviors	Sensitivity (%)	Specificity (%)	PPV (%)	NPV (%)
Residents and new inmates (*n* = 1892)				
Drug use, ever	95	31	9	99
Injecting drug use, ever	54	94	39	97
Shared injection equipment, ever	34	97	47	95
HCV screening, ever	43	69	9	94
Residents (*n* = 1482)				
Drug use, ever	96	30	10	99
Injecting drug use, ever	58	94	43	97
Shared injection equipment, ever	37	98	55	95
HCV screening, ever	49	63	10	94
New inmates (*n* = 410)				
Drug use, ever	89	32	6	98
Imprisonment, ever	89	32	6	98
Injecting drug use, ever	26	94	18	96
Shared injection equipment, ever	16	96	17	96
HCV screening, ever	0	91	0	95

*PPV* positive predictive value, *NPV* negative predictive value

**Table 6:** Characteristics of the questionnaire for detecting HCV antibody categorized by opioid agonist therapy (OAT) history

History of risk behaviors	Sensitivity (%)	Specificity (%)	PPV (%)	NPV (%)
People on OAT (*n* = 621)				
Drug use, ever	100	9	12	100
Injecting drug use, ever	65	90	46	95
Shared injection equipment, ever	45	95	56	93
HCV screening, ever	45	68	15	90
People with a history of OAT (*n* = 241)				
Drug use, ever	94	29	9	98
Injecting drug use, ever	39	93	29	95
Shared injection equipment, ever	25	97	36	95
HCV screening, ever	25	63	5	92
People with no history of OAT (*n* = 949)				
Drug use, ever	87	42	6	99
Injecting drug use, ever	39	96	29	98
Shared injection equipment, ever	17	98	29	97
HCV screening, ever	47	72	7	97

*PPV* positive predictive value, *NPV* negative predictive value

The sensitivity and negative predictive value of the other questions were low and ranged from 34 to 54% and 94 to 97% among all participants, respectively. Having a history of injection and sharing injection equipment was more sensitive for case finding among residents than new inmates (sensitivity: 58% vs. 26% and 37% vs. 16%, respectively), and people on OAT than those with no such history (sensitivity: 65% vs. 39% and 45% vs. 17%, respectively). The question about previous HCV testing did not find any new inmates with positive antibody but was 49% sensitive for case finding among residents. Among people on OAT, and those participants with and without a history of OAT, testing history was 45%, 25%, and 47% sensitive, respectively.

### Concordance of the risk-based questionnaire and RNA testing

The drug use question was the most accurate predictor of having a positive HCV RNA results as well (sensitivity: 96.7%, negative predictive value: 99.5%). The sensitivity and negative predictive value of injection and sharing questions among all inmates ranged from 59 to 63%, and 97 to 73%, respectively. Only 9% (8/90) of people who had positive HCV RNA results truly answered that they currently have HCV disease. From participants who responded that they have HCV disease currently, only 33% (8/24) had positive RNA results, while among people who believed they do not have the disease, 3% (6/205) tested positive.

## Discussion

This study validates a risk-based questionnaire for the identification of people with HCV infection in Gorgan central prison, Iran. Overall, 7.5% of residents and 4.6% of newly admitted inmates had a positive HCV antibody, which was a remarkably higher prevalence than the general population [5]. Participants who were currently receiving OAT had a higher HCV antibody prevalence, compared to those with no history of OAT (11.4% vs. 4.0%). The history of drug use was the most accurate predictor of having positive HCV antibody and RNA results, with respectively 95% and 97% sensitivity. This outcome indicates that in low- and middle-income settings, HCV screening for people with no history of drug use could be skipped in correctional facilities.

The HCV antibody prevalence in Gorgan prison was less than some previous Iranian reports, but fourteen times higher than the general population [7, 9]. Compared to a 2003 study, the prevalence of HCV antibody in this prison has decreased, which could be partly due to the implementation of harm reduction programs in recent years [20]. However, given the voluntary-based non-random design of our study, the results cannot be generalized. In Iran, HCV care models that are adapted for the specific needs of marginalized populations are emerging, including for people in custody; however, current interventions and harm reduction coverage are still not sufficient for breaking the cycle of transmission [21].

While the choice of a screening strategy is highly influenced by the expected prevalence and budget priorities, evidence on the different HCV screening approaches in low- and middle-income countries is scarce [18, 22]. In resource-limited settings, risked-based screening is postulated to be of value for case finding among target populations [7, 19]. However, the reliability of self-reported risk behaviors and the consistency with which the screening criteria are applied have shown to limit the early case detection ability [19, 23]. Studies on the efficacy of a risk-based HCV strategy among marginalized populations are limited, and to date, case finding by surveys have been only done in high-income countries [18, 19]. Previous studies have shown benefits for universal screening in correctional facilities, compared to the risk-based screening, which is in contrast to our findings [18, 24]. A possible explanation could be that in Iran, no taboo exists around non-injecting drug use among the incarcerated population, which results in more reliable self-reported information [25]. To avoid loss of treatment opportunity in prisons, the diagnosis and linkage to care for this hard-to-reach population should be obtained immediately upon admission [18, 26, 27]. Therefore, tailored screening strategies should be developed to scale-up diagnosis among people in custody.

Evaluation of the HCV screening among target populations in resource-limited countries is limited [28]. In this study, only 30% of the prison population had been tested for HCV before, from whom 59% were not aware of their test results, indicating the insufficiency of prison-based screening programs as well as poor HCV knowledge and post-test counseling. The history of HCV testing is similar to the previous estimates among people who use drugs in Iran (30% vs. 28%) [21]. However, compared to people attending substance use treatment programs in the USA, the rate of people who had never been tested for HCV in this prison was two-fold higher (69% vs. 30%) [29]. Besides, self-reported information regarding the previous HCV test results with a high rate of false positives and negatives was unreliable (67% and 3%), which is a common observation among prison studies [30].

The history of drug use among all participants was remarkably high and very similar to the results from a study on 6200 Iranian inmates (71% vs. 74%) [4]. The prevalence of drug use between residents and newly admitted inmates was almost similar (72% vs. 69%), which could be due to the fact that the majority of new inmates had a history of prior imprisonment (69%). Among all inmates with a history of drug use, 9% had a positive antibody that is 18-fold higher compared to the general population of Golestan province, where the study site is located [31]. Moreover, participants who were currently receiving OAT represented a more vulnerable population for HCV infection compared to those with no history of OAT (11.4% vs. 4.0%). These results indicate that future HCV screening efforts should focus on people with a history of drug use, particularly those who are attending or have a history of OAT. Comprehensive public health response to HCV in prisons should include tailored screening strategies and drug intervention programs such as OAT scale-up, together with assessment of health risk behaviors [21].

The history of drug use was the most sensitive question for predicting a positive HCV antibody test among all inmates (95%); this sensitivity was lower among newly admitted inmates compared to residents (89% vs. 96%), highlighting the fact that new inmates are more reluctant to disclose their drug use. Despite the high sensitivity of the drug use question, the other risk factors were low sensitive for case finding: among people with a positive HCV antibody test, 46% and 66% did not report any history of injecting drug use and sharing, respectively. Therefore, lifetime experience of injecting drug use and sharing needles is under-reported among people in prison. The unreliability of self-reported history of injecting drug use had been reported in other studies as well, which could be due to the existing reluctance of inmates to disclose their risk behaviors and face probable punishments in custody [32]. It seems that risk-based screening could become a replacement for universal HCV testing provided that ongoing efforts are made to reduce stigma around drug use and injection in societies. Further, despite the extensive harm reduction efforts in the recent decade, such programs are still inadequate in Iran, as well as many other countries [33].

The main strength of this study was its novelty in developing a low-cost strategy for countries with limited resources in the battle against HCV. However, our study had some limitations like using self-reported data, which its reliability has been questioned [19, 23]. The main limitation is that because of the high costs of HCV RNA screening for all inmates, we assumed that our antibody kit has almost 100% sensitivity for diagnosing people with HCV infection. Thus, having a negative antibody is considered as negative RNA testing, which may slightly change our results due to the testing bias. However, in a previous study, we have shown that the sensitivity of our rapid screening test is almost 100% [34]. Another limitation was that among newly admitted inmates with positive antibody (*n* = 19), 11 people were released before RNA testing. The venipuncture samples could not be collected daily, given limited capacity for RNA testing at the local laboratory and the lack of a fixed nurse in prison for obtaining blood samples. Participants were receiving HCV rapid testing upon admission daily, but those with a positive antibody were undergoing venipuncture after a few days, during which someone might be released. Aside from newly admitted inmates, all of the prison residents with positive antibody tests underwent venipuncture and had data available. Also, the small number of female participants limits the generalizability of this study.

## Conclusions

Screening for HCV infection based on having a history of drug use could replace universal screening in correctional facilities to reduce costs. However, the history of drug injection and sharing injection equipment remain under-reported. History of HCV testing is sub-optimal among inmates in Iran; therefore, effective prison-based programs are needed to scale-up HCV diagnosis and linkage to care for the people who are rarely reached by healthcare systems. Moreover, drug interventions and co-morbid health assessments should be enhanced to reduce the harms from people who cycle through custody. In resource-limited settings, tailored HCV screening strategies are crucial for pursuing elimination targets, and further cost-effectiveness analysis is needed to confirm the optimal strategies.

## Data Availability

The datasets used and/or analyzed during the current study are available from the corresponding author on reasonable request.

## Abbreviations

HCV: Hepatitis C virus
OAT: Opioid agonist therapy
LMIC: Low- and middle-income countries
WHO: World Health Organization
PWID: People who inject drugs
DAA: Direct-acting antivirals
TB: Tubercle bacillus
HIV: Human immunodeficiency virus
APRI: AST to Platelet Ratio Index
PPV: Positive predictive value
NPV: Negative predictive value
IQR: Interquartile range
